# Endocranial anatomy of the earliest Cretaceous European neosuchian crocodyliform *Pholidosaurus purbeckensis* provides new evidence for the ecological evolution of Pholidosauridae

**DOI:** 10.1111/joa.70125

**Published:** 2026-02-20

**Authors:** Leonardo Barbini, Paul M. J. Burke, Ilaria Caddeo, Marco Romano, Philip D. Mannion

**Affiliations:** ^1^ Dipartimento di Scienze Della Terra Sapienza Università di Roma Rome Italy; ^2^ Department of Earth Sciences University College London London UK

**Keywords:** computed tomography, Crocodylomorpha, endocast, endocranial anatomy, neuroanatomy, Pholidosauridae, *Pholidosaurus*, salt glands

## Abstract

The neosuchian crocodyliform clade Pholidosauridae had a near‐cosmopolitan distribution, spanning the Late Jurassic to the early Paleocene. Representatives of the group inhabited aquatic environments, ranging from freshwater to potentially fully marine forms. The phylogenetic placement of Pholidosauridae within Neosuchia remains debated: whereas most analyses place it as closely related to Dyrosauridae, some studies argue for a closer relationship with Goniopholididae. One skeletal region that could shed light on both the phylogenetic position of Pholidosauridae, as well as how it achieved its broad distribution, is the internal cranial anatomy, which has been shown to document morphological features with both an ecological and phylogenetic signal in other crocodyliforms. However, a natural endocast is currently the only available information on the internal cranial anatomy of a pholidosaurid. Here, we present new insights into the internal cranial anatomy of *Pholidosaurus purbeckensis*, based on CT‐scan data of material from the lowermost Cretaceous Purbeck Limestone Group, southern UK. Overall, the endocranial anatomy of *P*. *purbeckensis* is more similar to that of goniopholidids than dyrosaurids, especially the morphology of the olfactory tract and the cerebrum, which might represent a phylogenetic rather than ecological signal. However, this might merely reflect retention of the ‘standard’ crocodyliform skull and endocranial shape in pholidosaurids and goniopholids, rather than necessarily a close relationship, with the cranial anatomy of dyrosaurids instead representing an apomorphic departure from this morphology. We identify paired dorsolateral expansions in the olfactory region of the skull of *P*. *purbeckensis*, which have been interpreted as osteological correlates of nasal salt glands in some marine thalattosuchians, dyrosaurids, and extinct gavialoid crocodylians. If this interpretation is correct, it would suggest a higher tolerance for saltwater than previously hypothesised in *Pholidosaurus*, which would provide support for oceanic capabilities early in the evolution of Pholidosauridae that potentially enabled the group's near‐cosmopolitan distribution. Finally, we demonstrate external cranial anatomical variation amongst specimens attributed to *P*. *purbeckensis*, particularly in contemporaneous French remains provisionally referred to this species. However, this might best be regarded as individual variation: we therefore tentatively support the attribution of this material to *P*. *purbeckensis* pending the much‐needed revision of the type species of *Pholidosaurus*, *P*. *schaumburgensis*, from the earliest Cretaceous of Germany.

## Graphical abstract

We present new insights into the internal cranial anatomy of the neosuchian crocodyliform *Pholidosaurus purbeckensis*, based on CT‐scan data of material from the lowermost Cretaceous Purbeck Limestone Group, southern UK. From the study of the endocast, we obtained new information on the phylogeny and the ecological evolution of the Family Pholidosauridae.

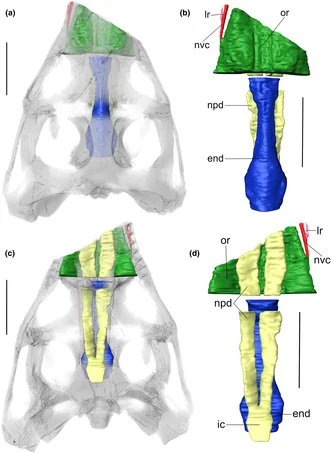

## 1 INTRODUCTION

Pholidosauridae is a clade of longirostrine neosuchian crocodyliforms that had a near‐cosmopolitan distribution, with their remains known from north Africa, western Europe, southeast Asia, and the Americas, spanning the Late Jurassic to the early Paleocene (Fortier et al., 2011; Jouve & Jalil, 2020; Martin et al., 2014, 2016). It therefore represents one of several crocodyliform lineages to have survived the end‐Cretaceous mass extinction (Jouve, 2021). Pholidosaurids inhabited aquatic environments, ranging from taxa recovered in freshwater ecosystems, including the gigantic Early Cretaceous north African species *Sarcosuchus imperator* (de Broin & Taquet, 1966; Sereno et al., 2001), through to potentially fully marine forms, such as *Oceanosuchus boecensis* and *Terminonaris* spp. from the Late Cretaceous of Europe and North America, respectively (Hua et al., 2007; Shimada & Parris, 2007; Wu et al., 2001). Fortier et al. (2011) suggested that the earliest pholidosaurids were probably adapted to a mixed environment, and members of the clade therefore potentially had the ability to migrate between freshwater and marine ecosystems (Martin et al., 2016), facilitating their cosmopolitan distribution (Martin et al., 2014).

In many recent phylogenetic analyses, pholidosaurids are recovered as close relatives of the Late Cretaceous–early Paleogene group Dyrosauridae (e.g. Jouve et al., 2006; Jouve & Jalil, 2020), which includes freshwater and fully marine longirostrine species from Africa, the Americas, and Europe (e.g. Hastings et al., 2011; Jouve et al., 2021), together forming the clade Tethysuchia (Andrade et al., 2011). However, whereas some of these studies recover a diverse Pholidosauridae (e.g. Fortier et al., 2011; Jouve & Jalil, 2020), others restrict it to only the type genus, *Pholidosaurus* (e.g. Andrade et al., 2011; Young et al., 2017) (see also Meunier & Larsson, 2017). A small number of phylogenetic analyses have instead recovered Pholidosauridae as more closely related to Goniopholididae (e.g. Martin et al., 2016), which consists of semi‐aquatic platyrostral species from the Jurassic–Cretaceous of Laurasia (Andrade et al., 2011), together forming the clade Coelognathosuchia (Martin et al., 2014; see also Buffetaut, 1982; Martin & Buffetaut, 2012). However, analyses supporting Coelognathosuchia have not included representatives of Dyrosauridae in the underlying dataset. By contrast, Groh et al. (2020) did not find evidence for either Tethysuchia nor Coelognathosuchia, but instead recovered Pholidosauridae, Dyrosauridae, and Goniopholididae as successively phylogenetically nested lineages. Most recently, Kuzmin et al. (2024) noted numerous differences between the braincase anatomy of pholidosaurids and dyrosaurids, which they suggested might further weaken support for Tethysuchia (see also Martin et al., 2016).

As the type genus for Pholidosauridae, *Pholidosaurus* is key to such debates. Currently, two species are typically considered valid by most authors: the type species, *P. schaumburgensis*, based on remains from the Berriasian (earliest Cretaceous) of Germany (von Meyer, 1841, 1846), and *P*. *purbeckensis* (Mansel‐Pleydell, 1888), which was erected based on several specimens from contemporaneous deposits from the UK, including partial skulls (Andrews, 1913; Jouve, 2017; Salisbury, 2002; Salisbury et al., 1999; Salisbury & Naish, 2011; Watson, 1911). Additional remains from the Berriasian of France, including a near‐complete skull, have since been referred to *P*. *purbeckensis* by Martin et al. (2016), with that study representing the only detailed anatomical description of either species to date.

Studies of the internal anatomy of the skulls of crocodyliforms demonstrate that they display a wealth of morphological information that can shed light on both the phylogenetic affinities and ecology of a species (e.g. Barrios et al., 2023; Burke et al., 2025; Ristevski, 2022; Schwab et al., 2020). However, a natural endocast reconstruction (Edinger, 1938) of a partial skull from the Berriasian of Germany that is probably referrable to *P. schaumburgensis* is currently the only available information on the endocranial anatomy of a pholidosaurid.

Here we present new information on the cranial anatomy of *P*. *purbeckensis*: for the first time, we provide an endocranial description of the species based on a virtual reconstruction via CT scanning. We utilise these new anatomical data to make comparisons with other neosuchian crocodyliforms, especially dyrosaurids, goniopholidids, and other pholidosaurids, including the French specimens assigned to *P*. *purbeckensis*. Finally, we also make interpretations of the palaeoecology of *P*. *purbeckensis* based on features of the endocranium.

*Institutional abbreviations*: CHV and TL, Collection Cherves‐de‐Cognac, Musée d'Angoulême, Charente, France; DORCM, Dorset County Museum, Dorchester, UK; NHMUK, Natural History Museum London, UK.

## 2 GEOLOGICAL SETTING

The Purbeck Limestone Group, well‐exposed in coastal sections of east Dorset, southern England, UK (Coram & Radley, 2021), records a Jurassic/Cretaceous transition from marine to non‐marine depositional conditions within the Wessex Basin (Underhill & Stoneley, 1998). It is subdivided into the Lulworth Formation and the overlying Durlston Formation (Casey, 1963; Townson, 1975), spanning the uppermost Tithonian to Berriasian (Coram & Radley, 2021; Hopson et al., 2008). The paleoenvironment of the Purbeck Limestone Group was highly dynamic, oscillating between hypersaline lagoonal settings and freshwater‐dominated systems (Coram & Radley, 2021). The Lulworth Formation exhibits substantial evaporite deposition, indicative of arid conditions with restricted marine influence. By contrast, the Durlston Formation represents a phase of increased freshwater influx, likely driven by enhanced precipitation and fluvial runoff from adjacent massifs: this marks a transition to increasingly brackish and freshwater‐influenced depositional environments, reflecting a shift in paleoclimatic and eustatic conditions (Coram & Radley, 2021).

The fossil record of the Purbeck Limestone Group is among the most diverse for non‐marine Berriasian successions globally (Coram & Radley, 2021). The type section at Durlston Bay, near Swanage, Dorset, has yielded a rich assemblage of invertebrates (molluscs, crustaceans, and insects), vertebrates (fish, amphibians, mammals, and reptiles), and flora (conifers, ferns, mosses, and charophytes) (Coram & Radley, 2021; Ensom, 2002; Martill et al., 2013; Milner & Batten, 2002). This includes referred specimens of *P*. *purbeckensis*, with NHMUK R36721, R3414, and OR28432 collected from quarries in the immediate vicinity of Swanage (Salisbury, 2002). Although the exact horizon from which they were recovered is uncertain, it is likely that they derive from the Intermarine Beds of the lower Durlston Formation (Salisbury, 2002), which represent a transition from a freshwater to increasingly brackish environment (Hopson et al., 2008; West, 1979; Westhead & Mather, 1996). The holotype of *P*. *purbeckensis*, DORCM G97, also comes from the Purbeck Limestone Group, although the stratigraphic horizon is unknown and it is uncertain as to whether it was collected from Swanage or the nearby Isle of Purbeck (Mansel‐Pleydell, 1888; Salisbury, 2002).

## 3 MATERIALS AND METHODS

Four specimens of *Pholidosaurus purbeckensis* (NHMUK R36721, NHMUK R3414, NHMUK OR28432 and NHMUK R3956), all representing partial skulls (Salisbury, 2002), were studied first‐hand. Anatomical information on the holotype skull, DORCM G97, was drawn from photographs provided courtesy of the Dorset Museum and Art Gallery. Comparisons with the two French skulls tentatively referred to *P*. *purbeckensis* (CHV03.100, TL) were based on information presented in Martin et al. (2016). NHMUK R36721 was scanned at the Natural History Museum, London, with X‐ray micro‐computed tomography, using a Nikon Metrology XTH 225 ST system (Nikon Metrology, Leuven, Belgium), with a reconstructed isotropic voxel size of 0.086 mm^3^. The internal cranial anatomy of NHMUK R36721 was subsequently segmented in Avizo v. 9.7 (FEI Visualization Science Group; https://www.thermofisher.com) by manually drawing the outline of the internal structures and then filling the area to define a selection. The selections of each slice were then interpolated to generate a three‐dimensional model of the endocast. This was done for every individual structure that was then identified with a different colour. Subsequently, the reconstructed regions were smoothed with the software Meshmixer (http://www.meshmixer.com). Morphometric data were collected from the endocast of NHMUK R36721 using the ‘Measurement’ tool in Avizo v. 9.7 and then compared with measurements from specimens of crocodyliforms presented in the published literature (Erb & Turner, 2021; Pierce et al., 2017) (Table 1). A 3D surface scan of NHMUK R36721 was also produced using an Artec Space Spider surface scanner (Artec 3D, Santa Clara, CA, USA; www.artec3d.com/portable‐3d scanners/artec‐spider‐v2), and was subsequently processed in Artec Studio 16 Professional software (Artec Group, Luxembourg, Luxembourg). All of the scan data are available via MorphoSource (DOI to be provided on acceptance).

**TABLE 1:** Comparative morphometric data of the endocranium and endosseous labyrinth of crocodylomorph taxa.

Measurements (mm)	*Pelagosaurus* (thalattosuchian)	*Plagiophthalmosuchus* (teleosauroid)	*Simosuchus* (notosuchian)	*Sebecus* (sebecid)	*Rhabdognathus* (dyrosaurid)	*Pholidosaurus* (pholidosaurid)	*Pholidosaurus* (pholidosaurid)	*Goniopholis* (goniopholidid)	*Gavialis* (crocodylian)	*Alligator* (crocodylian)	*Crocodylus* (crocodylian)
Skull width at cerebrum (b/w postorbitals) (SW)	52	?	58	147	89	136	?	?	168	73	?
Cephalic flexure angle (CF)	160	175	142	150	158	?	143	140	150	135	145
Pontine flexure angle (PF)	160	170	165	160	152	?	150	161	154	145	153
Endocast length (EL)	57	?	79	120	171	125	138	117	146	98	103
Olfactory tract length (+bulbs) (OL)	21	?	25	46	104	59	51	42	55	48	46
Cerebrum width (CW)	15	28	25	30	26	31	28	31	32	21	29
Pituitary width (PW)	6	14	5	?	5	?	12	15	6	5	5
Pituitary height (PH)	7	12	9	9	10	?	9	?	9	8	8
Pituitary length (PL)	10	17	10	8	14	?	20	?	11	10	11
Labyrinth height (LH)	14	26	?	?	25	?	?	?	21	18	13
Labyrinth width (LW)	11	26	?	?	26	?	?	?	21	14	14
Endosseous Cochlearduct length (ECL)	8	13	?	?	12	?	?	?	9	8	6
Anterior semicircularcanal area (AA)	9	38	?	?	19	?	?	?	36	35	18
Posterior semicircularcanal area (PA)	6	19	?	?	4	?	?	?	15	12	5
Lateral semicircularcanal area (LA)	4	14	?	?	8	?	?	?	22	13	8
Source	Pierce et al. (2017)	Brusatte et al. (2016)	Kley et al. (2010)	Colbert (1946) and Hopson (1979)	Erb and Turner (2021)	This study	Edinger (1938) and Hopson (1979)	Edinger (1938)	Pierce et al. (2017)	Witmer & Ridgely (2008)	Witmer et al. (2008)

*Note*: Question marks denote missing data. All measurements have been rounded to the nearest mm.

## 4 SYSTEMATIC PALEONTOLOGY

Mesoeucrocodylia (Whetstone & Whybrow 1983).

Neosuchia (Benton & Clark, 1988).

Pholidosauridae (Zittel & Eastman, 1902).

*Pholidosaurus* (von Meyer, 1841).

*Pholidosaurus purbeckensis* (Mansel‐Pleydell, 1888).

### 4.1 Holotype

DORCM G97, a nearly complete skull from the Purbeck Limestone Group (Berriasian) of the Isle of Purbeck or Swanage, Dorset, UK (Mansel‐Pleydell, 1888; Salisbury, 2002).

### 4.2 Referred specimens

NHMUK R36721, NHMUK R3414, NHMUK OR28432 and NHMUK R3956, partial skulls from the Durlston Formation, Purbeck Limestone Group (Berriasian) of Swanage, Dorset, UK.

## 5 DESCRIPTION

### 5.1 Preservation

**FIGURE 1:**
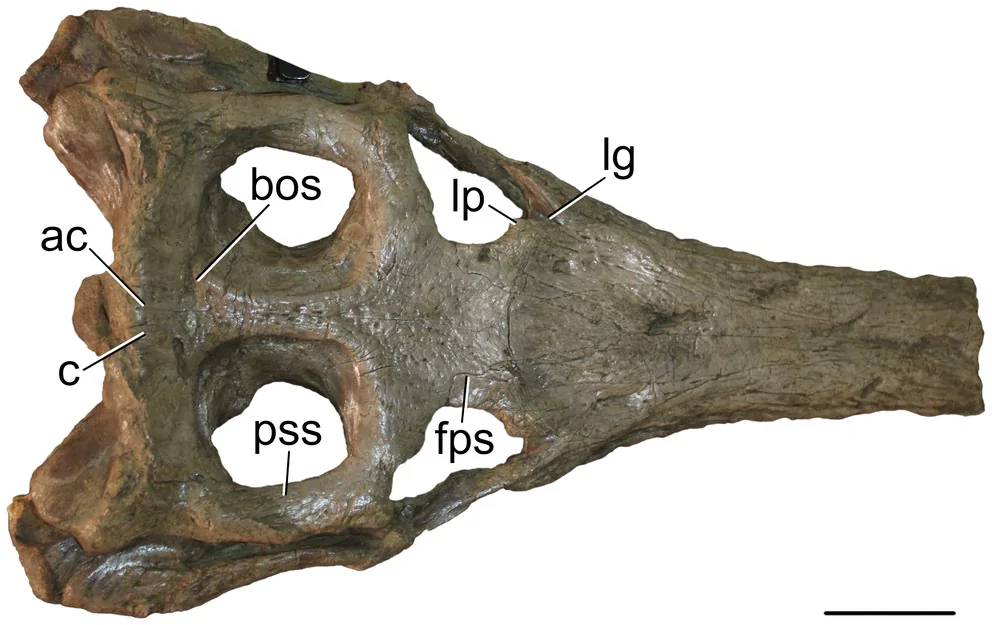
The holotype skull of *Pholidosaurus purbeckensis*, DORCM G97 in dorsal view, with matrix edited out. ac, anteroposterior crest; bos, break‐of‐slope on the parietal; c, concavity on the parietal; fps, frontal‐prefrontal suture; lg, lacrimal groove; lp, lacrimal prong; pss, postorbital‐squamosal suture. Photograph provided courtesy of the Dorset Museum and Art Gallery. Scale bar = 50 mm.

**FIGURE 2:**
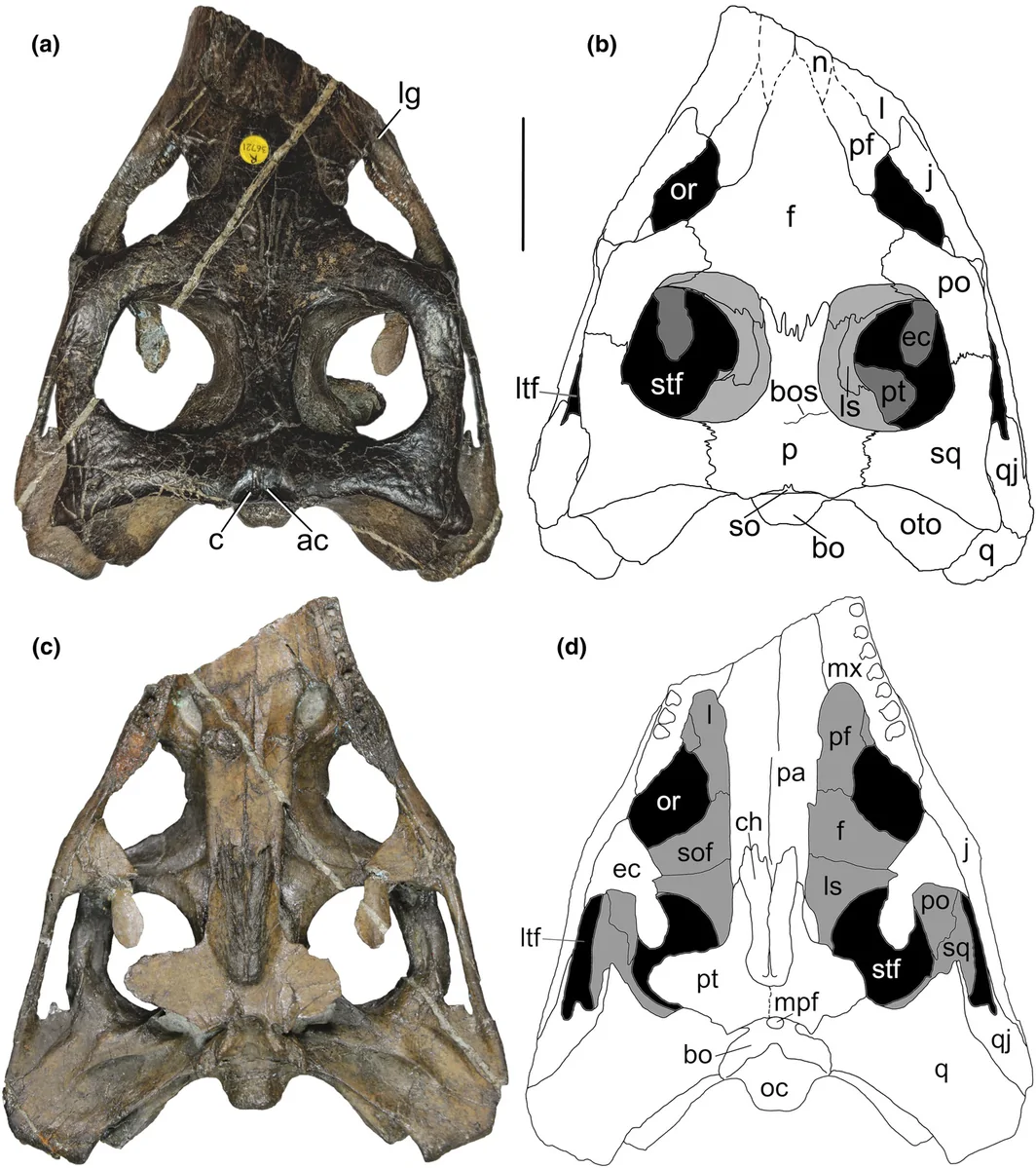
Referred skull of *Pholidosaurus purbeckensis*, NHMUK R36721. (a) Photograph in dorsal view. (b) Line drawing in dorsal view. (c) Photograph in ventral view. (d) Line drawing in ventral view. ac, anteroposterior crest; bo, basioccipital; bos, break‐of‐slope on the parietal; c, concavity on the parietal; ch, choanae; ec, ectopterygoid; f, frontal; j, jugal; l, lacrimal; lg, lacrimal groove; ltf, lower temporal fenestra; ls, laterosphenoid; mpf, median pharyngeal foramen; mx, maxilla; n, nasal; oc, occipital condyle; or, orbit; oto, otoccipital; pa, palatine; p, parietal; pf, prefrontal; po, postorbital; pt., pterygoid; q, quadrate; qj, quadratojugal; so, supraoccipital; sof, suborbital fenestra; stf, supratemporal fenestra; sq, squamosal. Dotted lines indicate uncertain sutures. Scale bar = 50 mm.

**FIGURE 3:**
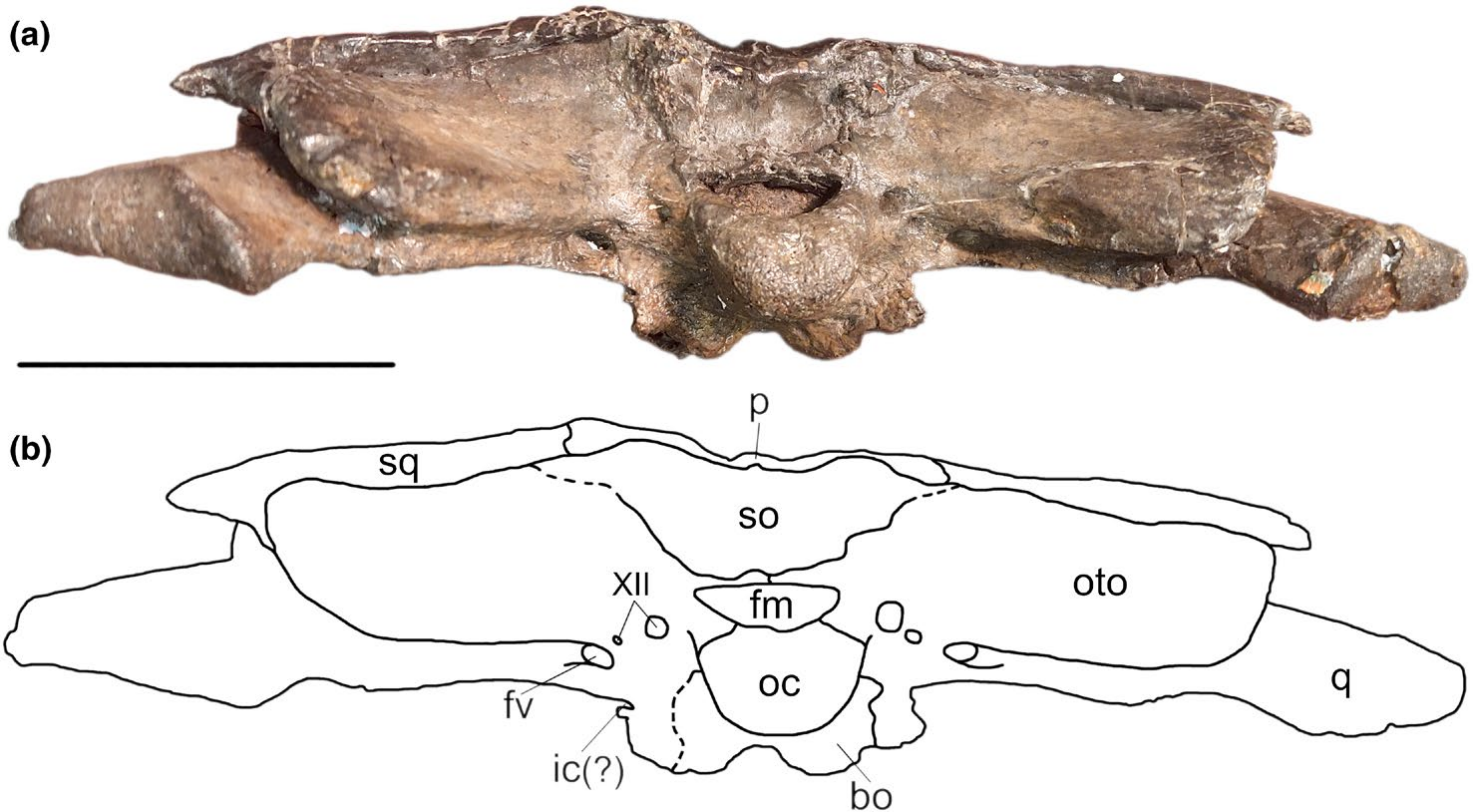
Referred skull of *Pholidosaurus purbeckensis*, NHMUK R36721. (a) Photograph in occipital view. (b) Line drawing in occipital view. bo, basioccipital; fm, foramen magnum; fv, foramen vagi; ic, internal carotid foramen; oc, occipital condyle; oto, otoccipital; p, parietal; q, quadrate; so, supraoccipital; sq., squamosal; XII, hypoglossal foramen (CN XII). Scale bar = 10 mm.

**FIGURE 4:**
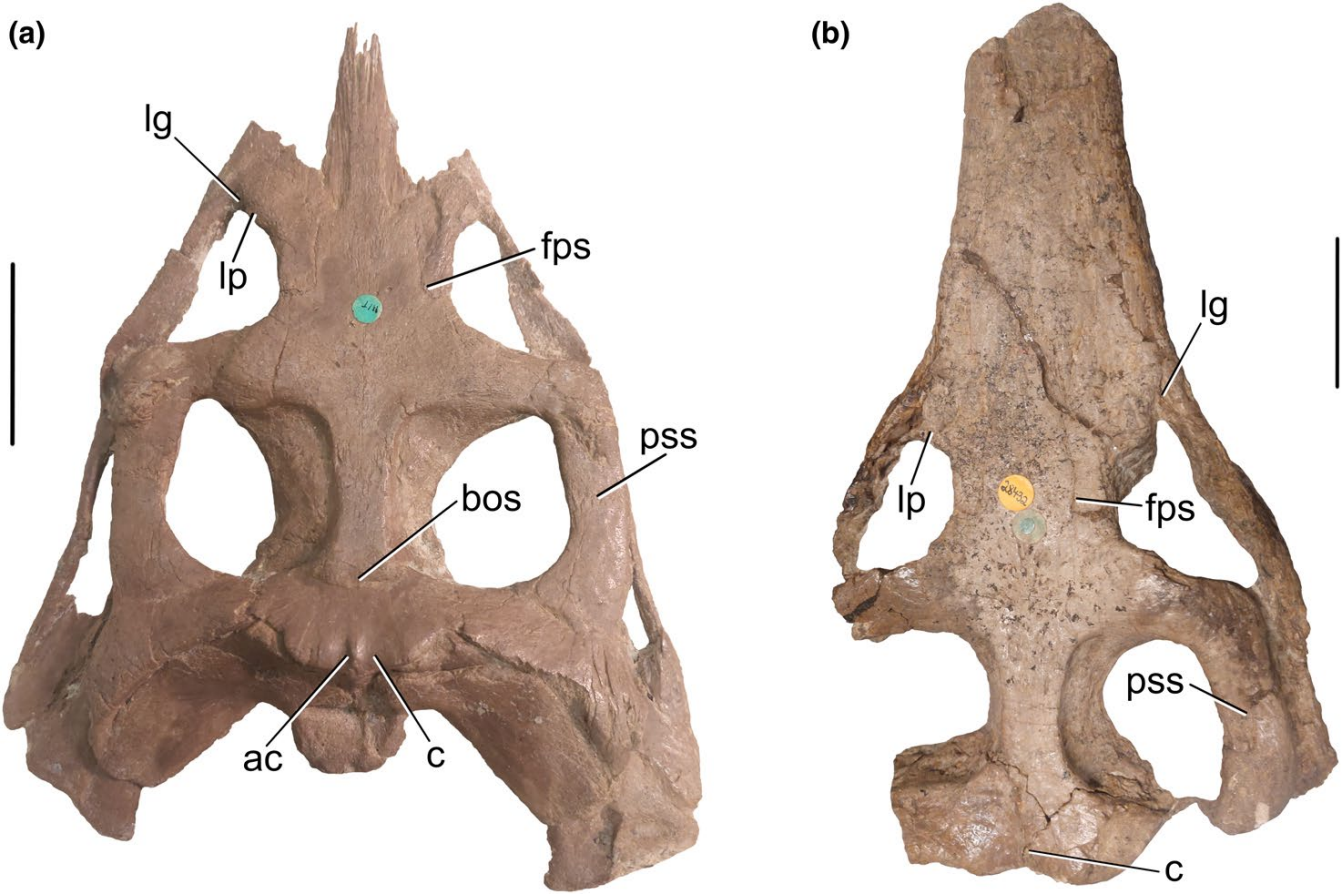
Referred skulls of *Pholidosaurus purbeckensis*. (a) Photograph of NHMUK R3414 in dorsal view, with matrix edited out. (b) Photograph of NHMUK OR28432 in dorsal view, with matrix edited out. ac, anteroposterior crest; bos, break‐of‐slope on the parietal; c, concavity on the parietal; fps, frontal‐prefrontal suture; lg, lacrimal groove; lp, lacrimal prong; pss, postorbital‐squamosal suture. Scale bars = 50 mm.

DORCM G97 is a near‐complete skull, lacking only the anterior tip of the snout. It is embedded in matrix, such that it is only possible to fully observe it in dorsal view (Figure 1). NHMUK R36721 (Figures 2 and 3) lacks almost all the snout, except the posteriormost part of the left side. The anterior part of the preserved skull is damaged, making it difficult to distinguish with confidence the sutures in this area. Some of the maxillary alveoli are visible, but no teeth are preserved. A major oblique fracture extends from the left quadrate to the right lacrimal. The specimen has also experienced strong dorsoventral compression that has affected both the external and internal morphology. As a consequence, the prefrontal pillars have penetrated the palatines. The pterygoids are also incomplete anteriorly. Otherwise, NHMUK R36721 is well preserved, and the sutures are clearly distinguishable across most of the skull. Both NHMUK R3414 and NHMUK OR28432 are embedded in matrix, such that they can only be observed clearly in dorsal view (Figure 4), and only partially in ventral view. NHMUK R3414 has experienced an even greater dorsoventral flattening than NHMUK R36721 and lacks all of the snout. NHMUK OR28432 is the least dorsoventrally flattened of the specimens studied in‐person and preserves the posterior part of the rostrum. The posterior part of the skull is also damaged, especially on the left side, where it lacks most of the postorbital and the squamosal. Although NHMUK R3956 can be considered the most complete of the NHM skulls, with a larger portion of the rostrum preserved, the specimen is currently mounted, such that neither the ventral surface, nor some other specific anatomical details can be assessed.

**FIGURE 5:**
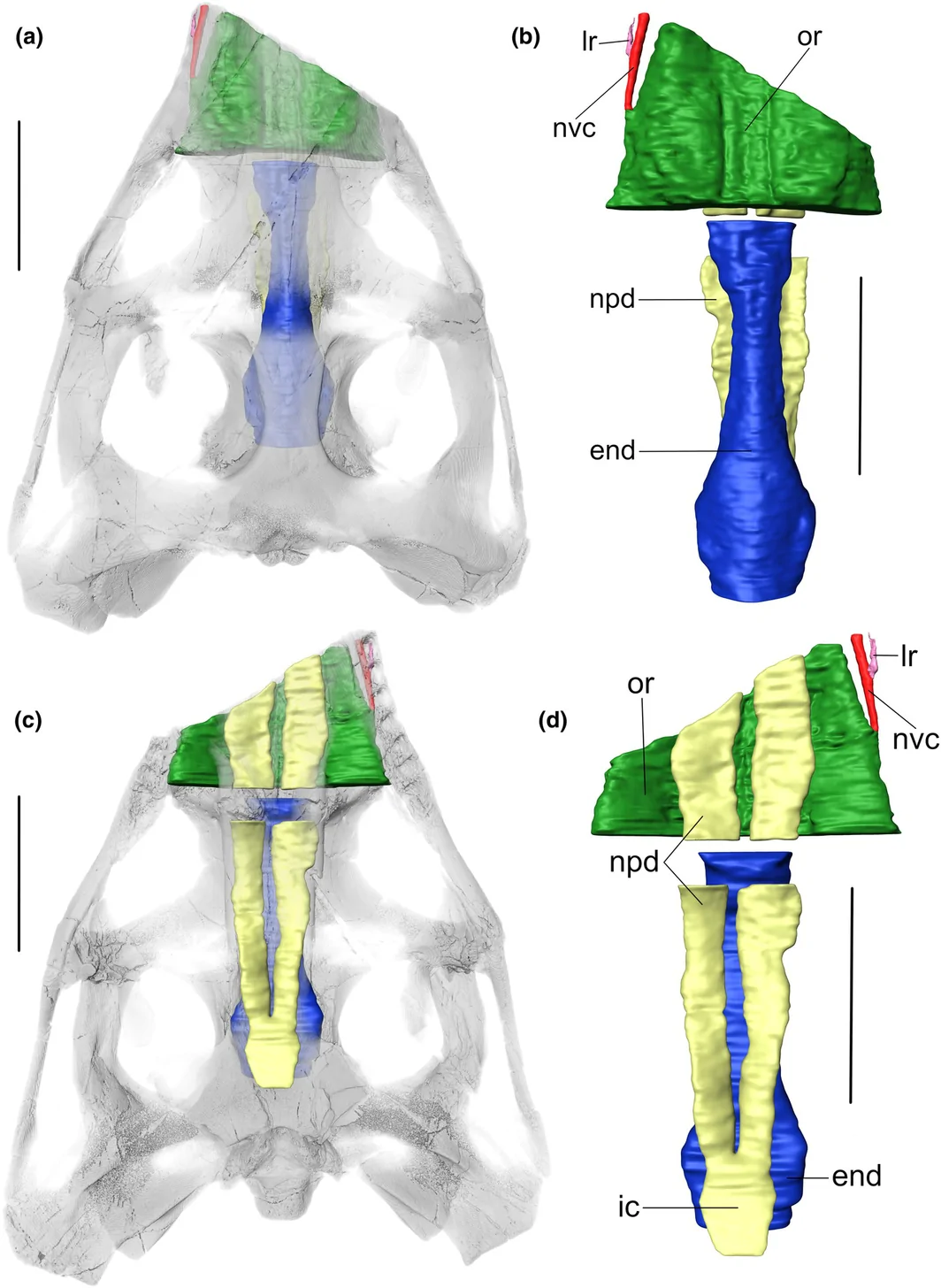
Endocast reconstruction of referred specimen of *Pholidosaurus purbeckensis* (NHMUK R36721). (a) Semi‐transparent 3D model with underlying endocranial anatomy, in dorsal view. (b) Isolated endocranial anatomy, in dorsal view. (c) Semi‐transparent 3D model with underlying endocranial anatomy, in ventral view. (d) Isolated endocranial anatomy, in ventral view. end, encephalic endocast; ic, internal choanae; lr, lateral ramification of the neurovascular canal; npd, nasopharyngeal duct; nvc, neurovascular canal; or, olfactory region. Scale bars = 50 mm.

**FIGURE 6:**
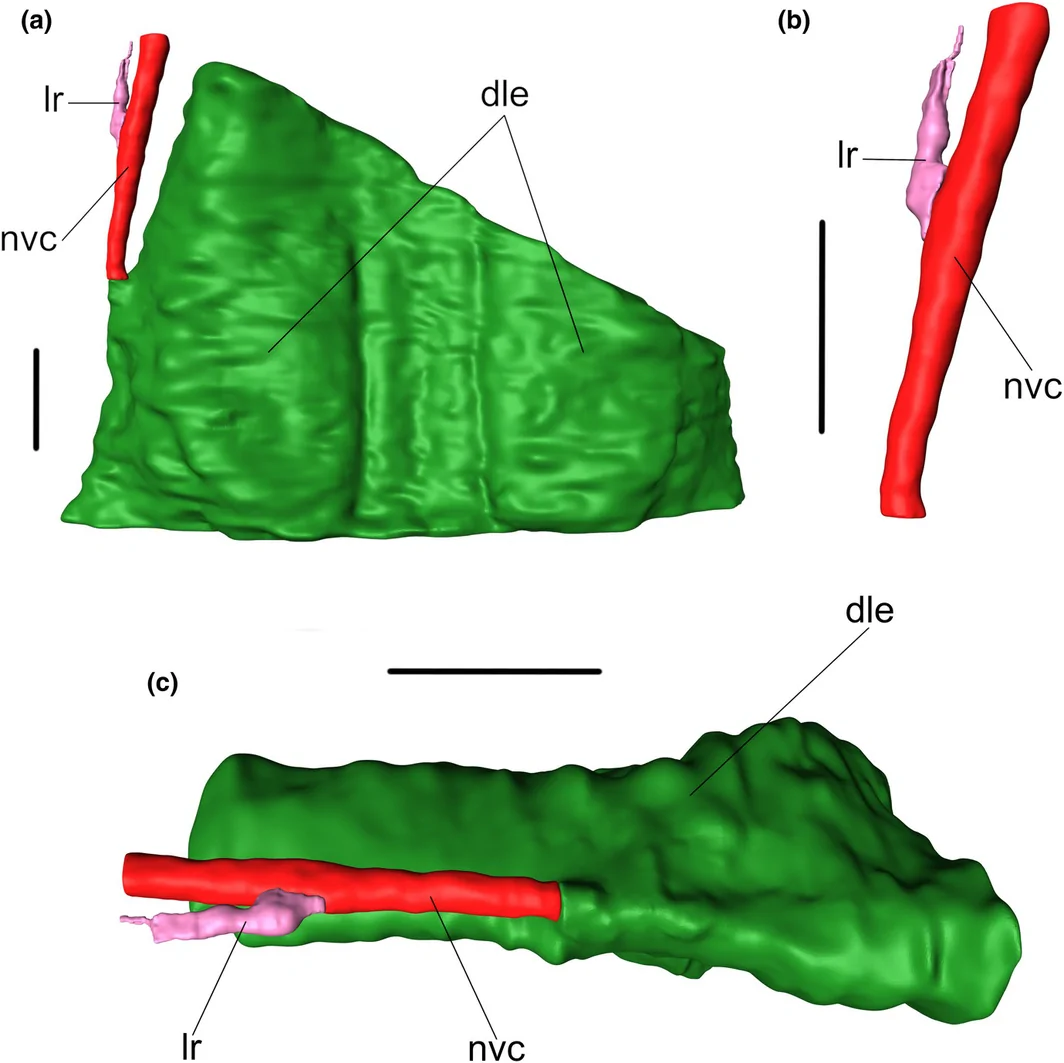
Reconstruction of the olfactory region of referred skull of *Pholidosaurus purbeckensis* (NHMUK R36721). (a) Dorsal view. (b) Close‐up of the neurovascular canal with its lateral ramification, in dorsal view. (c) Left lateral view. dle, dorsolateral expansions of the olfactory region; lr, lateral ramification of the neurovascular canal; nvc, neurovascular canal. Scale bars = 10 mm.

**FIGURE 7:**
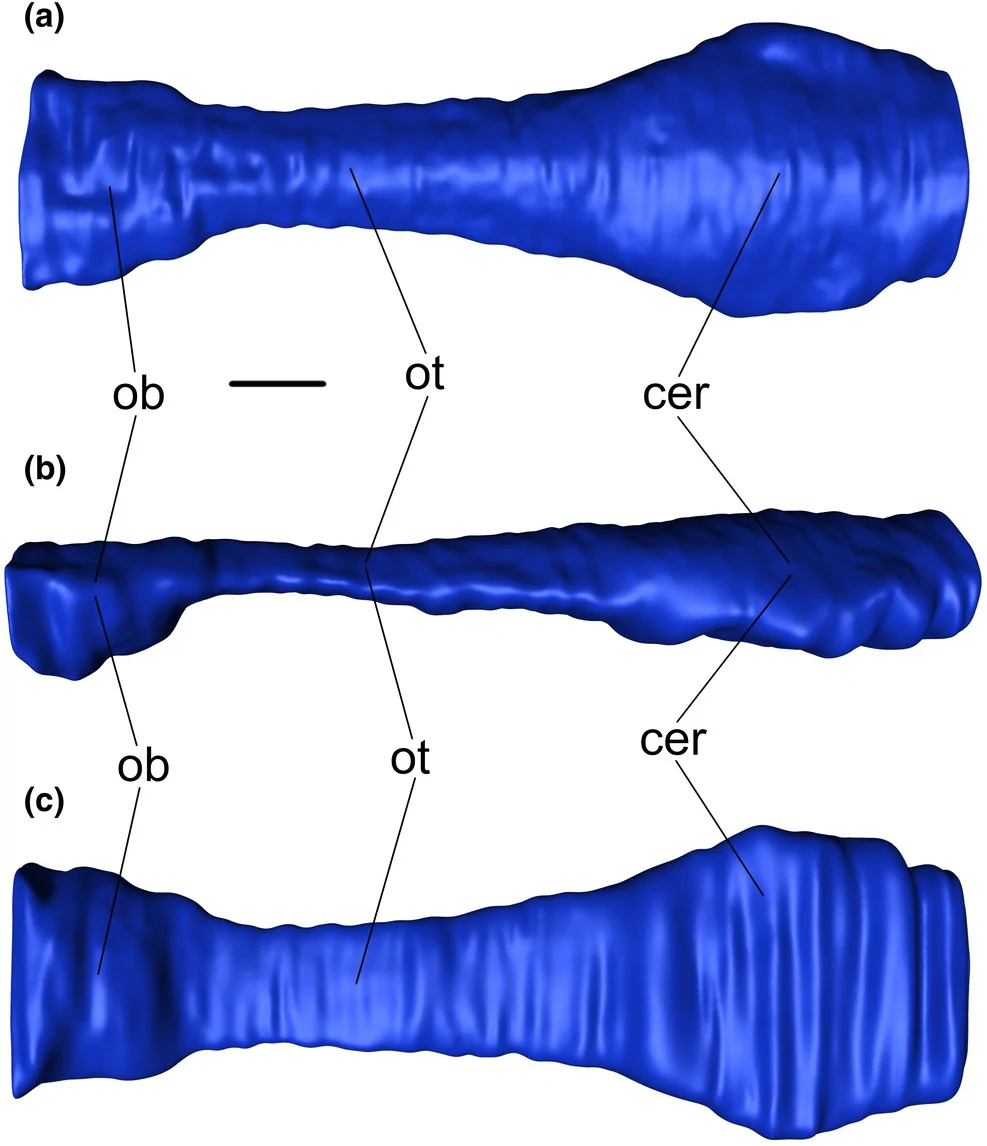
Encephalic endocast of referred skull of *Pholidosaurus purbeckensis* (NHMUK R36721). (a) Dorsal view. (b) Left lateral view. (c) Ventral view. cer, cerebrum; ob, olfactory bulb; ot, olfactory tract. Scale bar = 10 mm.

From the segmentation of the CT‐scan data of NHMUK R36721, it was possible to obtain a partial, though detailed, reconstruction of the endocast (Figure 5). The dorsoventral compression of the skull and the resultant ventral displacement of the prefrontal pillars means that there is breakage and corresponding gap in the reconstruction of the nasopharyngeal duct (Figure 5). The olfactory region is almost complete, only missing the anterior part on the right side (Figure 6a). A small gap has been left in our reconstruction of the transitional area between the olfactory region and the olfactory bulb because the area is not well preserved. As a result of poor internal preservation and or low fossil/matrix contrast, it was not possible to reconstruct the whole brain, with the endosseous labyrinths and paratympanic sinuses not visible at all. The brain cavity is visible only from its anteriormost part (the olfactory bulb) to just beyond the posterior end of the cerebrum, resulting in the complete absence of the cerebellum and the medulla oblongata (Figure 7). The strong dorsoventral flattening of the skull also resulted in breakage of the ventral portion of the braincase, with the inclusion of bone fragments in the cavity that are clearly visible in the 2D slices of the CT scans. Thus, the internal structures situated ventrally (e.g. the pituitary gland and the internal carotid arteries) could not be observed, and our reconstruction of this region in general has been affected by this compression.

### 5.2 Endocranial anatomy

#### 5.2.1 Nasal cavity and associated structures

**FIGURE 8:**
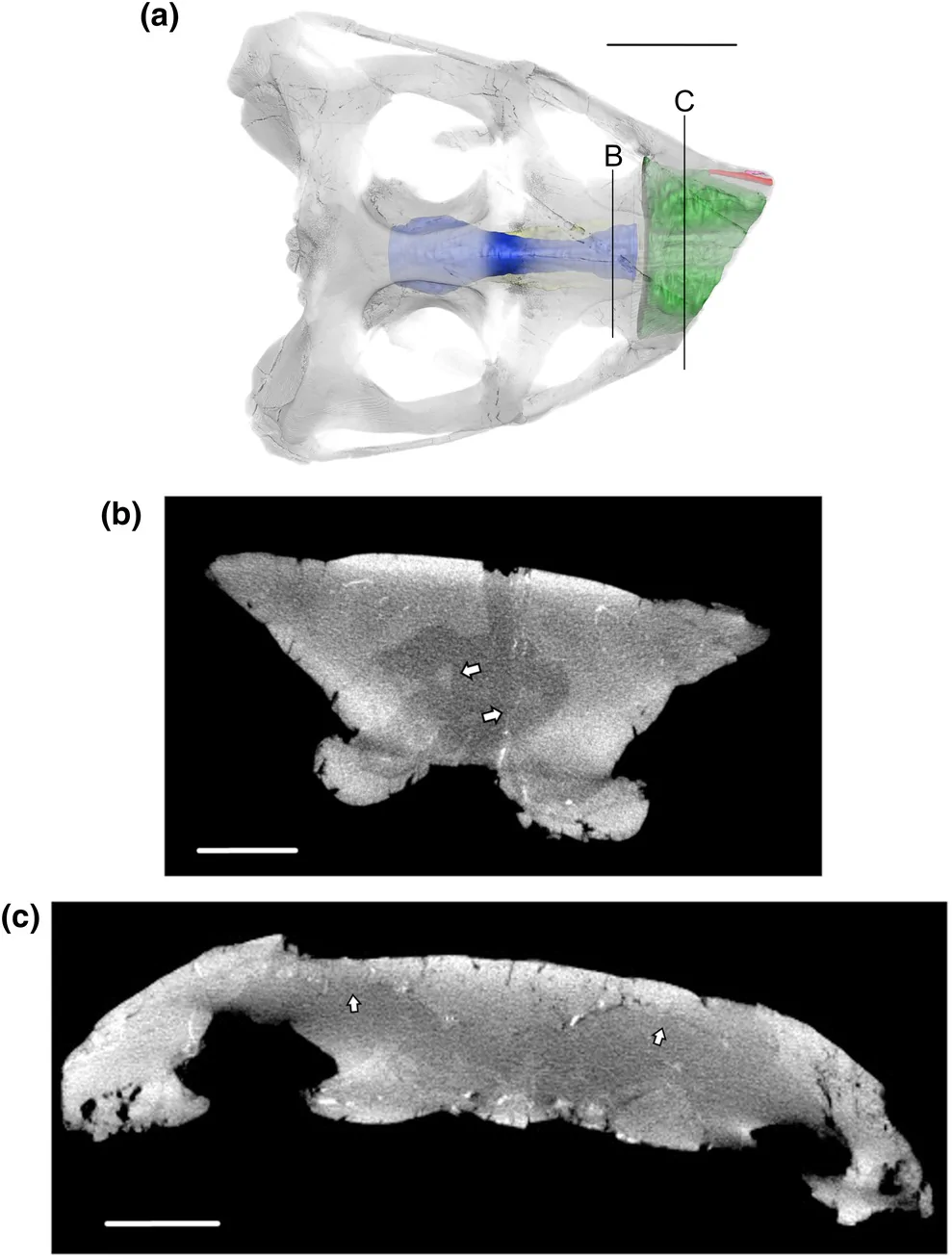
Endocast reconstruction of referred skull of *Pholidosaurus purbeckensis* (NHMUK R36721). (a) Skull in dorsal view, showing the position of the coronal CT slices. (b) CT slice of the olfactory bulb with detail of the two ‘bony processes’, highlighted by two white arrows. (c) CT slice of the olfactory region with detail of the dorsoventral expansions creating concave depressions on the internal surface of the prefrontals, indicated by two white arrows. Scale bars = 50 mm (a) and 10 mm (b, c).

**FIGURE 9:**
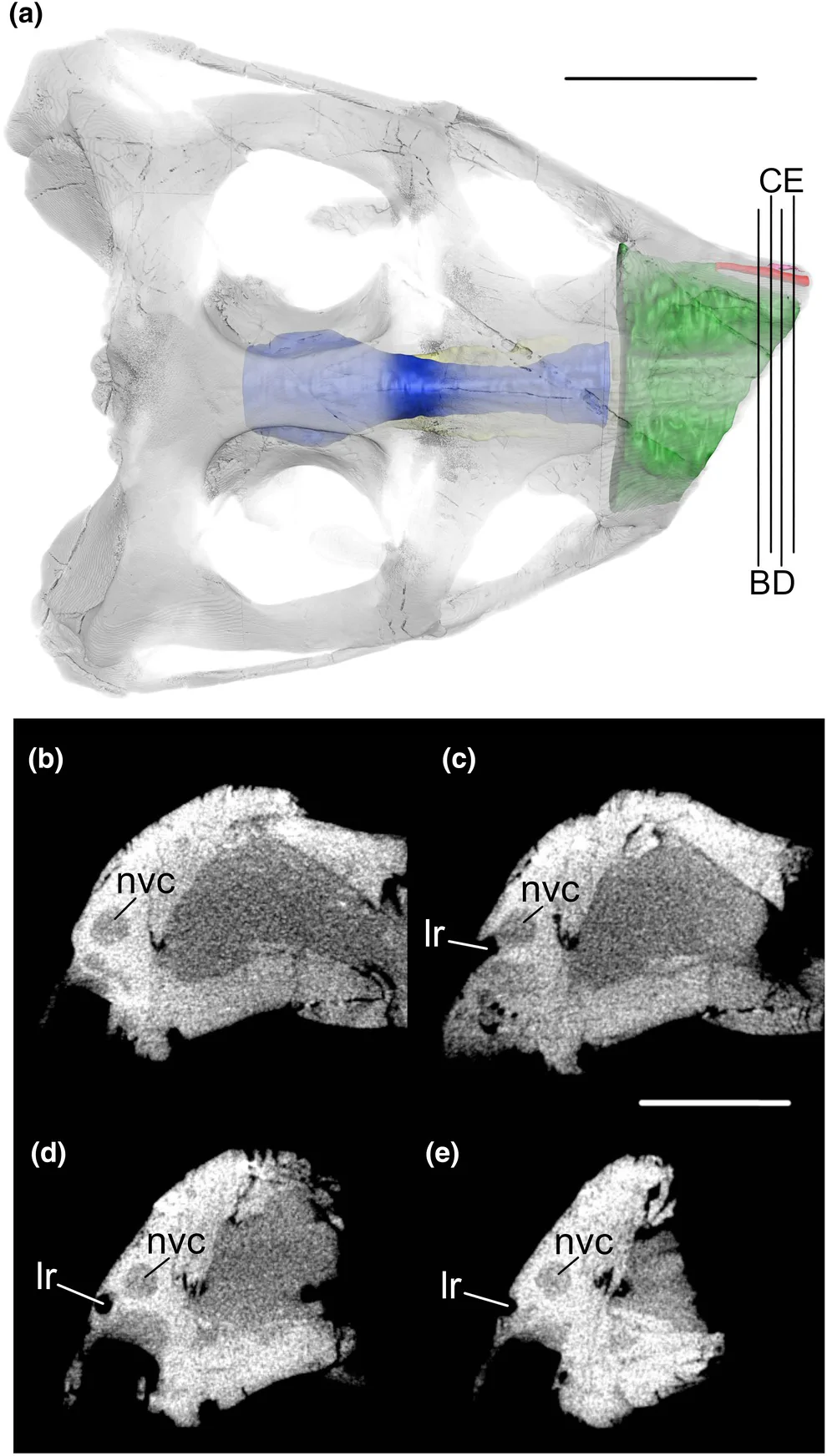
Endocast reconstruction of referred skull of *Pholidosaurus purbeckensis* (NHMUK R36721). (a) Skull in dorsal view, showing the position of the coronal CT slices. (b–e) CT slices of the olfactory region with detail of the neurovascular canal and its lateral ramification. lr, lateral ramification of the neurovascular canal; nvc, neurovascular canal. Scale bars = 50 mm (a) and 10 mm (b–e).

It is only possible to assess the posterior part of the nasal cavity proper (i.e. the olfactory region), the neurovascular canal on its left side, and the nasopharyngeal duct (Figures 5 and 6). The olfactory region bears a pair of bulbous dorsolateral expansions separated by a ridge on the ventral surface of the nasals. These two expansions result from concave depressions on the ventral surfaces of the prefrontals and the lacrimals (Figure 8c). Dorsal nasolacrimal ducts are either not present or could not be identified due to the poor preservation of this area. The preserved left neurovascular canal (=dorsal alveolar canal) runs lateral and parallel to the olfactory region (Figure 6b; see also Figure 9 for CT slices of the area): this would have housed the maxillary branch of the trigeminal nerve and the maxillary artery and vein (Pierce et al., 2017; Serrano‐Martínez et al., 2021). Approximately halfway along the segmented portion of this canal, a lateral ramification is present that runs parallel to the aforementioned canal: this is a passage that exits the skull at the level of the maxillary depression (see ‘External anatomical variability in specimens assigned to *Pholidosaurus purbeckensis*’ below). Ventral to the olfactory region, the nasopharyngeal duct consists of a pair of tubes and is visible up to the internal choanae.

#### 5.2.2 Endocranium

As in other non‐avian reptiles, the brain of adult crocodylians does not completely fill the endocranial cavity, mainly because of the presence of the dural envelope (e.g. Jerison, 1973; Hopson, 1979; Rogers, 1999; Hurlburt & Waldorf, 2002; Witmer et al., 2008; Brusatte et al., 2016; Jirak & Janacek, 2017; Hu et al., 2021; Barrios et al., 2023). Nonetheless, several portions of the brain can be observed, and information on their general shape and relative size can be obtained. Below it is implied that terms referring to soft tissue structures refer to their osteological correlates instead.

Overall, the general shape of the brain is consistent with the natural endocast of *Pholidosaurus schaumburgensis* described by Edinger (1938). The morphology of the endocast is dorsoventrally flattened as a consequence of the compression of the skull and because the pituitary gland and the other ventrally located structures could not be reconstructed (Figure 7). As the endocranium could only be partly reconstructed, it is not possible to provide exact values of the cephalic flexure (forebrain/midbrain) and the pontine flexure (midbrain/hindbrain), although the general shape of the brain can be confidently described as relatively straight. In the similarly straight endocast of *P*. *schaumburgensis*, the cephalic and pontine flexure angles are 143° and 150°, respectively (Barrios et al., 2023, table 7.2).

The olfactory bulb appears as an enlargement at the end of the olfactory tract, almost twice as wide mediolaterally as the tract and considerably expanded dorsoventrally. Two ‘bony processes’ seem to expand into the olfactory bulb, starting from the prefrontals (Figure 8b); however, it is likely that they represent fragments of broken bones derived from the breakage of the ventral portion of the skull. The olfactory tract connects the bulb to the rest of the brain and is undivided, anteroposteriorly long, but also mediolaterally wide relative to its length, with a width‐to‐length ratio of approximately 33%.

The cerebrum is a bulbous, laterally expanded structure that has its greatest expansion posteriorly. There is no sign of a midline groove between the cerebral hemispheres, such as observed in *Pelagosaurus typus* (Pierce et al., 2017), which reflects a thin dural envelope showing the underlying interhemispheric fissure, nor of a medial dorsal ridge, such as that present in *Rhabdognathus aslerensis* (Erb & Turner, 2021) and *Cricosaurus araucanensis* (Herrera et al., 2018), which reflects a well‐developed anterior portion of the dorsal longitudinal sinus.

### 5.3 External anatomical variability in specimens assigned to *Pholidosaurus purbeckensis*

The external morphology of the skull of *P*. *purbeckensis* is broadly consistent between DORCM G97 and the referred specimens, NHMUK R36721, NHMUK R3414, NHMUK OR28432, NHMUK R3956 and CHV03.100 (note that comparisons are not made with the second French skull, TL, because it is less complete and deformed [Martin et al., 2016]), but there are some differences. Below we outline anatomical variability amongst these specimens, as well as present some previously undocumented features of the external cranial anatomy of *P*. *purbeckensis*.

The ornamentation of the skull table consists of small sub‐circular pits and grooves, and its intensity is highly variable among the specimens. Both DORCM G97 and NHMUK R36721 present a well‐pronounced sculpturing, best developed on the parietal and the frontal (Figures 1 and 2). In the same area, NHMUK OR28432 and NHMUK R3956 show less pronounced ornamentation (Figure 4b), and in NHMUK R3414 the ornamentation is almost absent (Figure 4a), although in all three cases it is possible that this was affected by surface wear. However, all five UK specimens show a less marked sculpturing when compared with CHV03.100 (Martin et al., 2016, figure 2).

**FIGURE 10:**
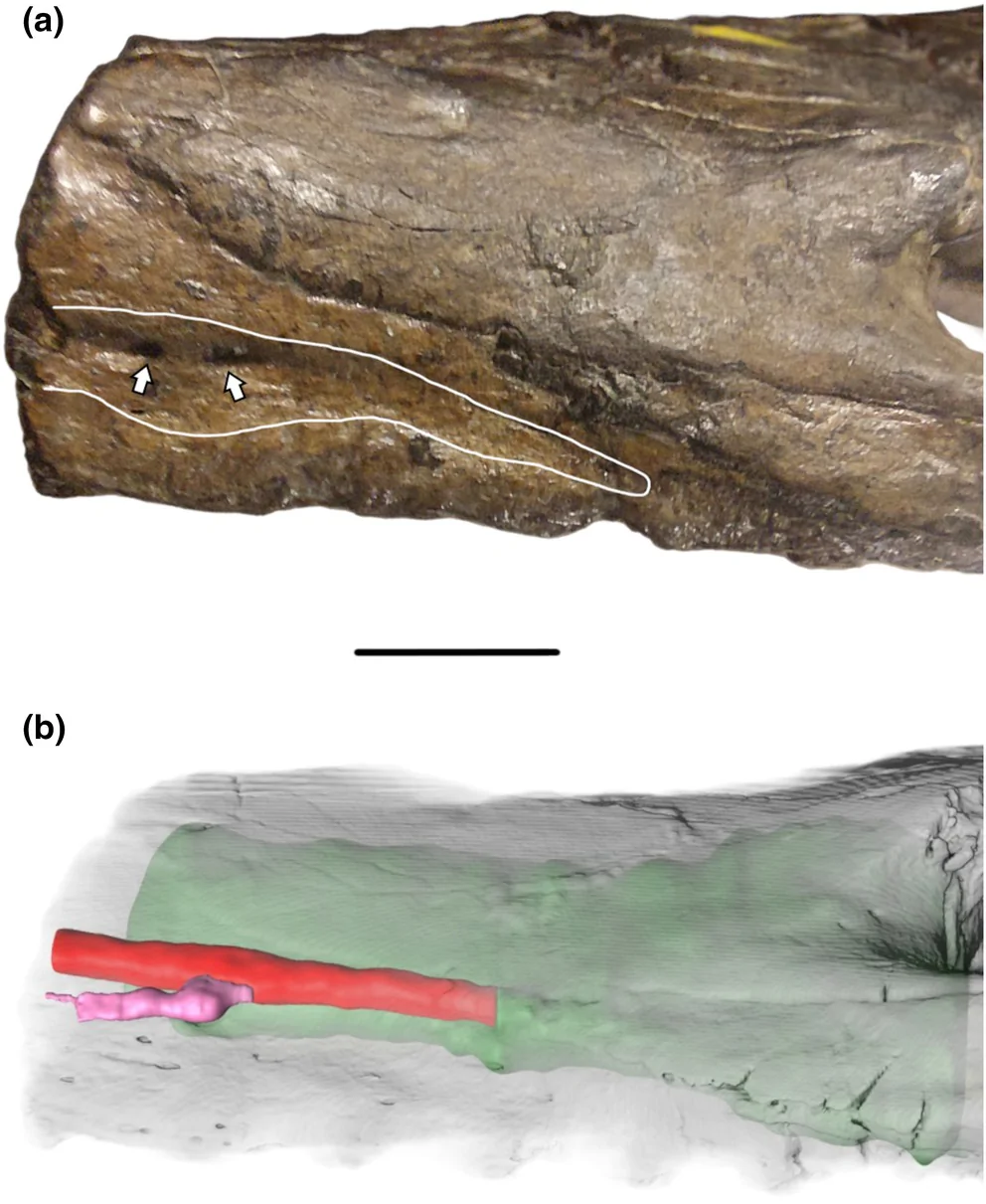
Left maxillary depression of referred skull of *Pholidosaurus purbeckensis* (NHMUK R36721). (a) Photograph in lateral view. The arrows indicate the channel‐like external opening. (b) Semi‐transparent 3D model with underlying endocranial anatomy. Green: Olfactory region. Red: Neurovascular canal. Pink: Lateral ramification of the neurovascular canal. Scale bar = 10 mm.

The presence of a maxillary depression, already highlighted by Martin and Buffetaut (2012) in CHV03.100 and the holotype of *P. purbeckensis* (DORCM G97), can also be confirmed in NHMUK R36721 (Figure 10). This shallow depression seems to be limited to the maxilla in DORCM G97 and NHMUK R36721, whereas it extends a few millimetres posterior to the maxillojugal suture in CHV03.100 (Martin & Buffetaut, 2012, figure 1e). The centre of the depression in NHMUK R36721 is pierced by a foramen that is set within a small, anteroposteriorly elongate channel‐like fossa that is walled by a thin portion of bone along its central third. This foramen is the external exit for the lateral ramification of the neurovascular canal described above. The presence of a foramen in this position was tentatively suggested in CHV03.100, but it has not been possible to assess in other specimens of *P. purbeckensis* (Martin & Buffetaut, 2012).

**FIGURE 11:**
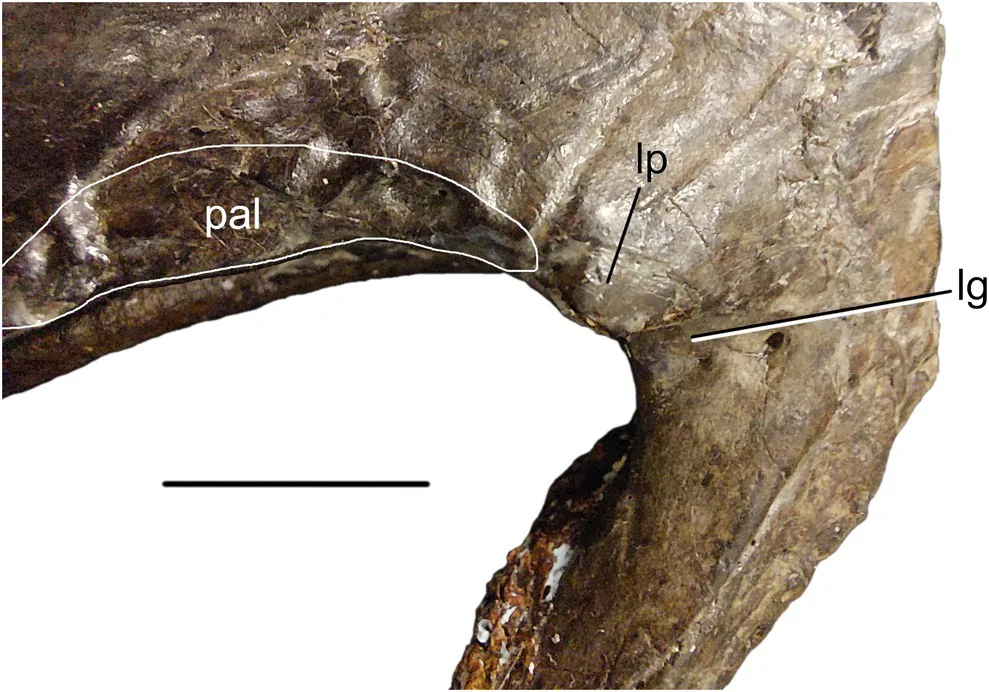
Right orbital region of referred skull of *Pholidosaurus purbeckensis* (NHMUK R36721). Photograph showing detail of the notch and the prong on the anterior margin, where the palpebral may have been accommodated. lg, lacrimal groove; lp, lacrimal prong; pal, palpebral notch. Scale bar = 10 mm.

A well‐defined notch on the prefrontal and an adjacent prong on the lacrimal is observed on the anteromedial margin of the orbit in DORCM G97 (Figure 1), NHMUK R36721 (Figures 2 and 11) and NHMUK R3956. This region is more difficult to assess in NHMUK R3414 and NHMUK OR28432, but a similar morphology can be identified on the left side of both specimens (Figure 4). A comparable lacrimal prong can also be observed in CHV03.100 (Martin et al., 2016, figure 2). Although no palpebral is preserved in any of the specimens, Salisbury and Naish (2011) suggested that this region might have housed a palpebral bone. Along the anterior margin of the orbit, a groove is present on the dorsal surface of the lacrimal of all UK specimens (Figures 1, 2, 4 and 11) but is seemingly absent in CHV03.100 (Martin et al., 2016, figures 2, 4 and 5). This lacrimal groove is similar to that of goniopholidids (e.g. Andrade & Hornung, 2011; Arribas et al., 2019; Ristevski et al., 2018).

Although it is incomplete in most of the analysed specimens, the mediolateral width of the anterior part of the frontal, relative to the interorbital width, appears to vary: it is greatest in NHMUK R3414 and NHMUK OR28432 (Figure 4), followed by DORCM G97 (Figure 1) and NHMUK R36721 (Figure 2), and smallest in CHV03.100 (Martin et al., 2016, figure 2). The suture between the frontal and the prefrontal is oriented mediolaterally along its posterior section and anteroposteriorly along the remainder of the suture, but the angle between these two sections is variable: in DORCM G97 and NHMUK R36721 this angle is slightly obtuse (Figures 1 and 2), with this angle slightly greater in CHV03.100 (Martin et al., 2016, figure 2), whereas it is close to 90° in NHMUK R3414 and NHMUK OR28432 (Figure 4). In the UK specimens, the frontal participates in the orbital margin to a similar extent as (or slightly less than) the prefrontal (Figures 1, 2 and 4), whereas the contribution of the frontal to the orbital margin is much smaller in CHV03.100 (Martin et al., 2016, figure 2).

The suture between the postorbital and the squamosal is situated approximately level with the anteroposterior mid‐length of the supratemporal fenestra in all five UK specimens (Figures 1, 2 and 4). By contrast, this suture appears to be situated more anteriorly in CHV03.100 (Martin et al., 2016, figure 2); however, the suture is indicated with a dashed line in Martin et al. (2016; figure 2), which might be indicative of uncertainty in its identification. As such it remains possible that the suture is in a similar position to that of the UK specimens.

The tip of the capitate process of the laterosphenoid does not reach the postorbital bar in CHV03.100 (Martin et al., 2016), whereas it does in NHMUK R36721. This feature cannot be assessed in detail in the remaining specimens since they are embedded in matrix and only observable in dorsal view; nevertheless, the capitate process appears to reach the postorbital bar in NHMUK R3414.

In DORCM G97, NHMUK R36721, R3956 and R3414, there is a break‐of‐slope on the dorsal surface of the parietal: anterior to this, the parietal is relatively flat, whereas it slopes ventrally level with the posterior margin of the supratemporal fenestrae, flattening again posteriorly (Figures 1, 2 and 4). This is more prominent in NHMUK R3414, although it is likely that this morphology is accentuated by, or possibly entirely resultant from, taphonomic deformation. A concave area is also present along the posterior margin of the dorsal surface of the parietal that is subdivided by a short anteroposteriorly oriented midline crest: this morphology is especially pronounced in NHMUK R36721 and R3414 (Figures 2 and 4), clearly observable in the holotype (Figure 1), and present but much less evident in NHMUK R3956. The relevant region in NHMUK OR28432 is damaged, preventing a clear assessment of the parietal morphology, but the concave area is clearly visible (Figure 4b). The concave area is also present in CHV03.100, but the crest dividing it, as well as the break‐of‐slope, seem to be absent (Martin et al., 2016, figure 2).

On the occipital surface of NHMUK R36721 (Figure 3), there is a small foramen situated ventrolateral to the opening for CN XII, which we herein interpret it as the opening for a second branch of the hypoglossal nerve. This additional opening is not present in CHV03.100 (Martin et al., 2016, figure 6) and it is not possible to assess the number of openings in the other four specimens. It is important to note that this feature has been shown to be intra‐specifically variable in at least some other crocodyliforms species (e.g. Brochu, 2007). The foramen vagi in NHMUK R36721 (Figure 3) is also much larger in diameter than that of CHV03.100 (Martin et al., 2016, figure 6).

## 6 DISCUSSION

### 6.1 Is the French material attributable to *Pholidosaurus purbeckensis*?

The two French specimens, CHV03.100 and TL, collected from the Berriasian site of Cherves‐de‐Cognac, Charente, were tentatively assigned to *Pholidosaurus purbeckensis* by Martin et al. (2016). Those authors noted that this referral remained provisional because comparisons could not be easily made with the type species of *Pholidosaurus*, *P*. *schaumburgensis*, from the earliest Cretaceous of Germany, the holotype of which consists of a natural mould of a partial vertebral column. Furthermore, the results of the phylogenetic analysis in Martin et al. (2016) do not support this referral, with the UK and French specimens occupying contrasting placements within Pholidosauridae, rather than forming a clade.

Our comparisons between the UK and French material highlight some additional morphological variability, which might cast further doubt on the assignation of the French remains to *Pholidosaurus purbeckensis*. However, most of these differences are subtle and at least some of the observed variation is greater within the UK specimens. As such, it could be indicative of intra‐ rather than inter‐specific variation. Furthermore, the phylogenetic analysis of Martin et al. (2016) was not a fair test of the relationships between the UK and French remains because it did not include anatomical features that potentially unite these specimens as characters. Therefore, pending a detailed revision of *Pholidosaurus schaumburgensis*, we follow Martin et al. (2016) in provisionally referring the French material to *Pholidosaurus purbeckensis*.

### 6.2 Internal anatomical cranial comparisons with other neosuchians, with a focus on Dyrosauridae and Goniopholididae

Although incomplete, the digital endocast reconstruction of *Pholidosaurus purbeckensis*, based on NHMUK R36721, as well as the natural endocast of *P. schaumburgensis* described by Edinger (1938), enable comparisons with other crocodyliform species. This includes representatives of both Goniopholididae and Dyrosauridae, that is the two clades considered to be the sister taxon to Pholidosauridae under competing phylogenetic hypotheses (e.g. Andrade et al., 2011; Martin et al., 2016), with published information available for a specimen of *Goniopholis* sp. from the earliest Cretaceous of the UK (Edinger, 1938) and *Rhabdognathus aslerensis* from the Paleocene of Mali (Erb & Turner, 2021).

With a width‐to‐length ratio of approximately 33%, the olfactory tract of *P. purbeckensis* is mediolaterally wider and anteroposteriorly shorter than the extremely narrow and elongate olfactory tract of the dyrosaurid *Rhabdognathus* (see Erb & Turner, 2021, figure 2). The morphology observed in *Pholidosaurus* is instead more similar to that of other neosuchians including *Paralligator* (Kuzmin et al., 2025) and the allodaposuchid *Agaresuchus* (Serrano‐Martínez et al., 2021), but especially to that of *Goniopholis* (Edinger, 1938, figure 2; Barrios et al., 2023, figure 7.9) which has an olfactory tract with the highest width‐to‐length ratio among the aforementioned taxa. However, it is possible that rather than necessarily indicating a close relationship, the similarity between pholidosaurids and goniopholidids reflects the plesiomorphic retention of the ‘standard’ crocodyliform skull and endocranial shape, with the extremely elongate olfactory tract of dyrosaurids instead representing an apomorphic departure from this morphology.

The cerebrum of *Pholidosaurus* has its greatest mediolateral expansion at its posterior end, remaining relatively narrow anteriorly. This morphology is similar to the cerebrum of *Goniopholis* (Edinger, 1938, figure 2; Barrios et al., 2023, figure 7.9), *Allodaposuchus* (Blanco et al., 2015) and *Paralligator* (Kuzmin et al., 2025), whereas the cerebral hemispheres are more symmetrical in *Rhabdognathus*, resulting in a nearly square morphology in dorsal view (Erb & Turner, 2021, figure 2).

Concave depressions in the prefrontals, similar to those found in thalattosuchians (Cowgill et al., 2023; Pierce et al., 2017), extinct gavialoids (Burke et al., 2025; Pligersdorffer et al., 2025), and now *Pholidosaurus*, have been hypothesised to also be present in *Rhabdognathus* (Pligersdorffer et al., 2025). However, their identification in the latter taxon was based only on the observation of four CT slices presented in Erb & Turner (2021, figure 3), and thus their presence should remain tentative. To date, published information on the endocranial anatomy of goniopholidids is limited to the brain cavity of *Goniopholis*; as such, it is not currently possible to determine whether or not these concave depressions were present in members of this clade. Although this feature might have a phylogenetic signal, uniting Pholidosauridae and Dyrosauridae, it is clear from its taxonomic distribution that it also has an ecological signal (see below), independently acquired in thalattosuchians and gavialoids; therefore, it might not necessarily be indicative of a close relationship of pholidosaurids and dyrosaurids to the exclusion of goniopholidids.

Although we consider it most likely that the two ‘bony processes’ inside the olfactory bulb of NHMUK R36721 (Figure 8b) are portions of broken bone emanating from the ventral region of the skull, similar processes are also present in at least some dyrosaurids, namely *Congosaurus* and *Rhabdognathus* (Erb & Turner, 2021; Jouve & Schwarz, 2004). However, even if we were not to consider the ‘bony processes’ in *Pholidosaurus* as a preservational artefact, the homology of these structures in the two clades would be doubtful: whereas in *Pholidosaurus* the processes extend from the prefrontals, they extend from the frontal in *Rhabdognathus*, and are probably formed by the frontal or an ethmoidal ossification in *Congosaurus* (Erb & Turner, 2021; Jouve & Schwarz, 2004). From the natural endocast of *Goniopholis* described by Edinger (1938), which is the only source of information on the endocranial anatomy of the taxon, no equivalent structure can be identified. However, since a natural endocast is less likely to preserve such small features, this absence might also be a preservational artefact.

In summary, these comparisons indicate a greater morphological similarity between the internal cranial anatomy of pholidosaurids with goniopholidids than dyrosaurids. Although this might reflect a broader similarity to most crocodyliforms, rather than necessarily a close relationship between the two groups, this might ultimately provide greater support for Coelognathosuchia rather than Tethysuchia. However, a greater number of species of each clade need to be assessed and these morphological features need to be incorporated into a well‐sampled phylogenetic dataset of neosuchians to properly test this hypothesis. Furthermore, we still lack detailed information on features such as the endosseous labyrinth and paratympanic sinus system of *Pholidosaurus* (some of these regions are partially preserved in the endocast described by Edinger, 1938, but are not very informative) and Pholidosauridae more broadly: these anatomical regions could provide essential insights into the phylogenetic relationships and ecology (see below) of pholidosaurids.

### 6.3 Palaeoecological implications

The UK remains of *Pholidosaurus purbeckensis* derive from the Purbeck Limestone Group. The exact stratigraphic horizon is uncertain, but it seems likely that most (if not all) of the specimens emanate from the Intermarine Beds of the Durlston Formation (Salisbury, 2002), which represents a transition from a freshwater to increasingly brackish environment. Although the French specimens referred to *P. purbeckensis* were found in a hypersaline lagoonal environment, they have been interpreted as allochthonous, with the remains hypothesised to have been transported from a nearby freshwater environment (Martin et al., 2016; Pouech et al., 2014). Our study of the skull of *P*. *purbeckensis* has uncovered some anatomical details that can potentially help infer the ecological habitat of this species, including how it might have been able to disperse between these regions during the earliest Cretaceous, with implications for Pholidosauridae more broadly.

If the ‘bony processes’ invading the olfactory bulb in NHMUK R36721 are not a preservational artefact, they might have had an ecologically relevant function in *Pholidosaurus*. Erb and Turner (2021) suggested that a similar structure in *Rhabdognathus* might have increased surface area for olfaction, as a potential coastal‐feeding adaptation for improved prey identification. Laterally placed orbits in *Pholidosaurus* might also be related to increased visual acuity in an aquatic pursuit predator, as previously suggested for the thalattosuchian *Pelagosaurus* (Pierce et al., 2017; Pierce & Benton, 2006).

As described above, a neurovascular canal runs lateral to the olfactory region in *Pholidosaurus*, with a ramification that reaches the external surface of the skull via a foramen in the centre of the maxillary depression. Amongst Crocodyliformes, a maxillary depression is only found in Goniopholididae and Pholidosauridae, being considerably larger and deeper in the former group (Martin & Buffetaut, 2012). A similar morphology has been described in other crocodyliforms, including *Hsisosuchus* (Gao, 2001) and *Shartegosuchus* (Dollman et al., 2018); however, the identification of this as a homologous structure is uncertain in both taxa (Dollman et al., 2018; Martin & Buffetaut, 2012). In goniopholidids, a sensorial function has been hypothesised, with the depression interpreted as an area of densely packed dome pressure receptors (DPRs), i.e. integumentary sensory organs present on the skin surface (Andrade, 2009; Martin & Buffetaut, 2012). DPRs in living crocodylians are innervated by the trigeminal nerve and are sensitive to pressure differences in the air–water interface, being an important detection and capture tool during ambush predatory behaviour (Andrade, 2009; Soares, 2002). It is possible that the foramen found in the middle of the maxillary depression of *Pholidosaurus purbeckensis* therefore represents evidence for some kind of trigeminal‐based sensory system, similarly to that hypothesised for goniopholidids.

Observed for the first time in a pholidosaurid, the paired, well‐developed dorsolateral expansions in the olfactory region of *Pholidosaurus* are similar to those of some thalattosuchians, including *Pelagosaurus*, *Eoneustes*, and *Cricosaurus*, in which they have been suggested to be an osteological correlate for the presence of hypertrophied antorbital salt glands (Cowgill et al., 2023; Pierce et al., 2017). These have also been interpreted to be present in some extinct gavialoids (Burke et al., 2025; Pligersdorffer et al., 2025), as well as the dyrosaurid *Rhabdognathus* (Pligersdorffer et al., 2025). The dorsolateral position of these expansions in *Pholidosaurus* (based on NHMUK R36721) is most similar to that of thalattosuchians, especially *Pelagosaurus* and *Eoneustes* (see Cowgill et al., 2023, figure 4). By contrast, the expansions are positioned more medially in the olfactory region in gavialoids (and probably dyrosaurids), resulting in a dorsal, rather than dorsolateral, positioning (Burke et al., 2025; Pligersdorffer et al., 2025).

The presence of these dorsolateral expansions in the olfactory region of *Pholidosaurus* might therefore indicate that nasal salt glands, similar to those found in metriorhynchoid thalattosuchians, were present; however, *Pholidosaurus* lacks the ‘preorbital fenestra’ that has been hypothesised to drain the excess salt in metriorhynchoids (Fernández & Herrera, 2009; Herrera et al., 2013). The extant marine iguana from the Galápagos Islands, *Amblyrhynchus cristatus*, possesses salt glands on the internal surface of its prefrontals and it excretes salt via its external nares (Dunson, 1969); it is possible that a similar mechanism existed in *Pholidosaurus* if this taxon truly possessed nasal salt glands. A similar drainage system at the level of the nasal vestibule has been also hypothesised for the small nasal salt glands inferred in teleosauroid thalattosuchians, which lack the ‘preorbital fenestra’ of metriorhynchoids (Cowgill et al., 2023).

In summary, the available evidence suggests that *Pholidosaurus purbeckensis* lived in freshwater and brackish environments and was probably capable of inhabiting saltwater environments too. Some mid‐Cretaceous pholidosaurids, such as *Terminonaris* and *Oceanosuchus*, have been recovered from marine depositional environments, suggesting an adaptation to coastal to oceanic habitats (Adams et al., 2011; Buffetaut & Wellnhofer, 1980; Hua et al., 2007; Wu et al., 2001). Hua and Buffetaut (1997) proposed a similar ecological lifestyle between *Terminonaris* and *Crocodylus porosus*, the extant saltwater crocodile, which can inhabit both freshwater and hypersaline waterways because of its lingual salt glands (Cramp et al., 2008). Our interpretation of an osteological correlate for salt glands in an early member of Pholidosauridae could indicate saltwater tolerance more widely within the clade, which might have been key to facilitating the group's near‐global distribution. Future studies should assess the internal cranial anatomy of other pholidosaurid taxa to test this hypothesis.

## 7 CONCLUSIONS

We present the first three‐dimensional reconstruction of the internal cranial anatomy of a pholidosaurid crocodyliform based on a specimen of *Pholidosaurus purbeckensis* from the earliest Cretaceous of the UK. Overall, its endocranial anatomy is more similar to that of goniopholidids than dyrosaurids; incorporation of this information into future phylogenetic analyses might help resolve the inter‐relationships of these three neosuchian clades. For the first time, we identify possible osteological correlates for the presence of salt glands in a pholidosaurid. This discovery suggests a higher degree of saltwater tolerance in this group than previously assumed. Such an adaptation may have facilitated the widespread distribution of Pholidosauridae during the Cretaceous, including the occupation of marine environments. External cranial anatomical variation amongst specimens attributed to *P*. *purbeckensis* is tentatively regarded as individual variation, but revision of the type species of *Pholidosaurus*, *P*. *schaumburgensis*, is needed to help distinguish between inter‐ and intra‐specific variation.

## AUTHOR CONTRIBUTIONS

LB designed the project, collected and analysed the data, segmented NHMUK R36721, led the writing of the manuscript, and produced the figures. PMJB assisted with the segmentation process, produced the 3D surface scan, and contributed to revising the manuscript. IC assisted with data collection and analysis and contributed to revising the manuscript. MR contributed to writing and revising the manuscript. PDM conceived and co‐designed the project and contributed to writing and revising the manuscript.

## Data Availability

The data that support the findings of this study are openly available in Morphosource at https://www.morphosource.org/.
